# Miscellaneous Items

**Published:** 1883-04

**Authors:** 


### EFFECT OF COMFORT ON THE QUALITY OF THE MILK.

The quiet and comfort of the cow has much to do with the quality of her milk. In hot weather, the annoyance produced by flies, and excitement caused by fighting them, makes the night's milk still poorer than it otherwise would be. Chemical analysis has shown a great falling off of fat in the milk of the same cow when chased by a dog. Any unusual excitement of the cow affects the fat in her milk. Extremes of heat and cold also affect the milk. In a case where cows went into a stream in hot weather, and stood several hours in the water above the knee, there was a falling off of the butter product from the same quantity of milk. This is accounted for by the extra food required to keep up the animal heat, in consequence of the heat being carried off by the water.

When we consider the fact that milk is secreted from the blood, we can readily see the effect that must be produced by excitement on the nervous system of the cow. In a case occurring in the city of Albany, N. Y., where a nervous cow was milked by a passionate man, who whipped and otherwise ill-treated her at milking, the milk was given to a child who had been healthy, but, after using this milk, became ill and suffered from intestinal irritation, followed by a fever which seemed to affect the brain and nervous system. This illness was traced directly to the milk of this ill-treated cow.—*Nationa Live Stock Journal.*

What to Do.—If a man faint, place him flat on his back, loosen his clothing, and let him alone.

Apoplexy is a sudden loss of consciousness without a suspension of breathing or circulation. The body becomes limp, as if there was no life. Place the patient on his back, with the head and shoulders slightly elevated; loosen his cravat and collar and the clothing about the chest, and then send for a physician.

If your Clothing takes Fire.—Your life may depend upon your good sense; slide the hands down the dress, keeping them as close to the body as possible, at the same time sinking to the floor by bending the knees; roll over on the floor and if possible get hold of a rug or tear up the carpet and envelope yourself in it. Nothing so quickly quenches the fire as to smother it. It may be done with a coat or bed clothing, or effectually by tearing up a carpet promptly and enveloping the person in it.

### ANÆSTHETIC BULLETS.

A German chemist has invented a new kind of bullet which, he urges, will, if brought into general use, greatly diminish, if not altogether remove, the horrors of war. The bullet is of a brittle substance, breaking directly it comes in contact with the object at which it is aimed. It contains a powerful anæsthetic, producing instantaneously complete insensibility, lasting for twelve hours, which, except that the action of the heart continues, is not to be distinguished from death. A battle-field where these bullets are used will in a short time be apparently covered with dead bodies, but in reality merely with the prostrate forms of soldiers reduced for the time being to a state of unconsciousness. While in this condition they may, the German chemist points out, be carefully packed n ambulance wagons and carried off as prisoners. Whole cities may in like manner be reduced to helplessness by means of shells charged with the same compound. The anæsthetic bullet is also strongly recommended to the burglar and to the householder, no risk of hanging being involved by its use.—*St. James' Gazette.*

---

Lake Michigan, which is 360 miles in length, and over 100 miles in breadth, would float the three States of New Jersey, Delaware and Maryland ; and it is deep enough anywhere to bury Mt. Holyoke, Mass., beneath its surface.

Prof. Sumner says the whole philosophy of wages was stated in a few words by a workingman a few years ago, and no economist can improve upon what he said: "I know when two bosses are running after one man wages are high ; when there are two men running after the one boss wages are low;" that embraces the science, theory and practice of the whole subject.

"Draw near thine ear, I pray thee," said Noah, as he sat smoking his good clay pipe by the fire, after having fed the animals their evening meal and shaken up their bedding. "What would my lord ?" replied Mrs. N., drawing near her ear, as commanded. Noah smoked in silence for the space of a minute or two, and then opened his mouth and spake as follows : "I perceive by the indications, that the storm which was central over the Euphrates will move westerly to the Nile Valley on the morrow, with areas of low barometer and northeasterly winds, and showery weather on the Arabian coast. I shall therefore get up steam at once and make for Ararat."

